# Explained and unexplained inter‐center variability in outcomes: Where should we go next?

**DOI:** 10.1111/ppe.13134

**Published:** 2024-10-27

**Authors:** Prakesh S. Shah

**Affiliations:** ^1^ Department of Pediatrics Mount Sinai Hospital and University of Toronto Toronto Ontario Canada

1

Variation in patient outcomes between different institutions, regions and countries has been reported across nearly all fields of medicine. However, in most cases, a large proportion of variance remains unexplained. Leaving institutions uncertain about how to act on such information. Moreover, in some instances, these differences are ‘accepted’ as natural variations and the system moves on without effort to reduce the variability.

In this issue of *Paediatric and Perinatal Epidemiology*, Mimura and colleagues^1^ report substantial variability in bronchopulmonary dysplasia rates in preterm neonates born at 22–31 weeks of gestation. They used an administrative database from 132 neonatal intensive care units, encompassing 11,496 neonates in Japan born between years 2014 and 2016. The authors identified that the rate of bronchopulmonary dysplasia had a median rate of 31.7% (interquartile range of 19.2%–44.9%), median odds ratio between the highest unit and the lowest‐performing units of 2.49, with an interval odds ratio of 0.19–6.23 and a proportion odds ratio of 47% (indicating that nearly half of the hospitals had odds ratios that were in opposite direction than overall odds ratio). These figures in essence indicate substantially high variability between units. The authors attempted to evaluate whether variation could be explained by hospital factors alone (by inserting hospital as a random intercept in the model), or by addition of patient factors, or by including management strategies (epulmonary surfactants, postnatal steroids, cyclooxygenase inhibitors, inhaled nitric oxide and respiratory support on day 6). They found minimal change in the median odds ratio and interval odds ratio across these models, suggesting that much of the variation remains unexplained.^1^

Variations in neonatal practices and outcomes have been reported in various networks and regions.^2^, ^3^ An international report, which included units from Japan, revealed marked variations in respiratory practices, particularly initial modes of ventilation. For example, most Japanese centers used mechanical ventilation as the first mode of respiratory support for neonates of <29 weeks' gestation, with 80% of the units using pressure‐controlled ventilation and 12% using high‐frequency ventilation. Other variations included oxygen saturation targets,^4^ surfactant administration, weaning strategies from respiratory support and respiratory medications like systemic or inhaled steroids between and within countries.^5^ Another factor associated with bronchopulmonary dysplasia is the treatment of patent ductus arteriosus. Routine treatment of asymptomatic ductus arteriosus was standard in 85% of Japanese units but not practiced in the remaining 15% of units.^6^ Hospital‐level variation in neonatal units also showed differences in neonates per room: 60% of units had 9–16 neonates/room, 20% of units had more than 16 neonates/room and only 3 units had a single‐room‐per‐baby design.^7^ In Japanese neonatal units, a senior neonatologist was present 24/7 in two‐thirds of the units (the highest among all surveyed countries); however, only one‐third of the units reported having nursing care assignments that were appropriate to the clinical status of each neonate.^8^ Thus, the reasons for variation likely include patient‐level, institution‐level, practice‐related, human, or system‐related factors. However, in many instances, the variation remains unexplained.^3^

Two aspects of this report deserve careful attention. First, what is the best method to assess variability between units or regions? Several methods are available to evaluate variability between units, regions, or countries. These include multilevel models incorporating various levels of characteristics (patient, unit, regional, health system levels), and reported as adjusted standardised ratios, using funnel plots, employing the empirical Bayes method, the propensity score method and fixed and random effect models, among others. Each of these techniques has its strengths and weaknesses. Many papers, including the current one in this journal, employ a combination of methods to ensure that the results are both comprehensive and robust.^1^

In most cases, multilevel hierarchical models and the empirical Bayes method are particularly effective because they can handle hospital volume and characteristics and provide hospital‐specific estimates in addition to accounting for patient‐level differences. Moreover, these methods offer uncertainty estimates around performance measures. Visualising these differences with funnel plots usually provides robust and interpretable insights for healthcare providers at the unit or hospital level. However, it is essential to remember that the central premise of such evaluations rests on the data's certainty and validity, the system's context and the outcome's significance. One might also wonder whether these models should be repeated at regular intervals to capture any changes resulting from shifts in patient demographics or the implementation of new initiatives.

Second, what are the reasons for these differences? Most of the methods mentioned above can account for measured variables. However, a large portion of unexplained confounding or variance often remains. Unexplained variance may arise from genetic, environmental, racial, ethnic, socioeconomic or other differences for which data may or may not be available. The overarching goal of a healthcare system is to ensure favourable outcomes for all patients and to reduce variability in outcomes between institutions. Waiting for a perfect explanation or a complete depiction of all sources of variance will continue to increase variability and inequity.

2

An important aspect of learning about and understanding variation is gaining valuable insights into what is or will be needed to address it. The data from such analyses can help initiate quality improvement activities, develop policies or guidelines that can be applied across units, and serve as a foundation for further research and innovation.^9^ In the specific context of a region or country, this information can also support the education and professional development of care providers, facilitate detailed cost‐effectiveness analyses and aid in the accreditation and certification of units. More advanced uses of this data may include the distribution and allocation of resources. In one way or another, identified variations in outcomes or processes must lead to meaningful changes in the healthcare system, particularly when wide variations are uncovered.

Several examples exist in the literature where system‐wide improvements were initiated based on identified variability between units or hospitals. For instance, the Northern New England Cardiovascular Disease Study Group^10^ employed transparent data reporting, a collaborative approach with a willingness to learn, continuous monitoring and timely feedback. These efforts led to a significant reduction in mortality rates following coronary artery bypass surgery in the region. Similarly, the Michigan State Surgical Quality Collaborative^11^ used risk‐adjusted outcome data to identify high‐performing hospitals. By sharing best practices and implementing evidence‐based interventions, they achieved an 18% reduction in surgical site infections and a 15% reduction in all postoperative complications in participating units. The ‘Evidence‐Based Practice, Identification and Quality (EPIQ)’ improvement initiative^12^ by the Canadian Neonatal Network has led to continuous improvements in neonatal and neurodevelopmental outcomes for preterm neonates born before 29 weeks' gestation over the past 15 years. Likewise, the Vermont Oxford Network's Quality Collaborative^13^ has reduced central line‐associated bloodstream infections, chronic lung disease and other complications in participating neonatal intensive care units.

These examples highlight several common themes: transparent data sharing, the use of risk‐adjusted outcome measures for comparison, the identification of high‐performing units, the implementation of evidence‐based practices, continuous monitoring and feedback, collaborative learning, the sharing of best practices, the engagement of multidisciplinary teams and, most importantly, the development of a culture of continuous quality improvement within units or regions are keys to effect a system change. Japan's national network of neonatal units engages in continuous quality improvement and results from this effort^1^ should spark a renewed and concerted action from all involved units, with the overarching goal of reducing variability and improving outcomes.

## ABOUT THE AUTHOR

**Prakesh S. Shah** is a Professor in the Department of Pediatrics and Institute of Health Policy, Management and Evaluation at the University of Toronto. He completed his medical and postgraduate training at Gujarat University, Ahmedabad, India and obtained further neonatal subspeciality training in the UK and Canada. He also completed his Masters in Clinical Epidemiology at the University of Toronto. He is the Director of Canadian Preterm Birth Network (CPTBN) and International Network for Evaluation of Outcomes of Neonates (iNeo).

## CONFLICT OF INTEREST STATEMENT

No conflict of interest to declare. The author is supported by grant funding from the Canadian Institutes of Health Research for the Canadian Preterm Birth Network (PBN 150642) and the International Network for Evaluation of Outcomes of Neonates (iNeo).
